# Supplementary material for: Antibacterial Agents Adsorbed on Active Carbon: A New Approach for S. aureus and E. coli Pathogen Elimination
Source: Pathogens. 2021 Aug 22;10(8):1066. doi: 10.3390/pathogens10081066 (PMC8401764; doi:10.3390/pathogens10081066)
Supplement: Supplementary file 1 [file pathogens-10-01066-s001.zip › pathogens-1287765-supplementary.pdf]

Supplemental Information

Antibacterial Agents Adsorbed on Active Carbon: A New Approach for Wound Healing Treatment

Ewa Burchacka [1, *], Katarzyna Pstrowska[1], Elżbieta Beran[1], Hanna Fałtynowicz[1], Katarzyna Chojnacka[1] and Marek Kułażyński [1]

[1] Department of Chemistry, Wroclaw University of Science and Technology, Wyspiańskiego Str. 27, 50-370 Wroclaw, Poland
[*] Correspondence: ewa.burchacka@pwr.edu.pl

*Corresponding Author: ewa.burchacka@pwr.edu.pl

Keywords: adsorption, active carbon, antibacterial treatment, antibiotics

Analytical part

The standard curve for the gentamicin (G)-ninhydrin complex

The standard curve for the sulfamethoxazole (S)

Supplemental figures

Figure S1 The microscopy picture of the area on PAC surface selected to elemental content analysis

Figure S2 The results of the content of elements for individually selected areas 1-3 and EDS spots 1-3

Figure S3 The standard curve for the gentamicin (G) - ninhydrin complex

Figure S4 The standard curve for the sulfamethoxazole (SMZ)

Figure S5 Release of gentamycin sulfate from PAC under aqueous conditions

Supplemental tables

Table S1 The results of intelligent eZAF calculations of the content of elements for individually selected areas 1-3 and EDS spots 1-3

Table S2 Filtration effectiveness of control samples: bacterial cultures *E. coli* and *S. aureus* and activated carbon PAC with LB medium.

Analytical part

The standard curve for the gentamicin-ninhydrin complex

Accurately 100 μg of gentamycin sulfate powder (Sigma-Aldrich CAS: 1405-41-0, Burlington, MA, United States) was weighed and dissolved with 1 mL of phosphate-buffered saline (PBS) solution (pH 7.4) to get the stock solution with the concentration of 100 μg/mL. Then dilutions of gentamicin sulfate were prepared in the range from 50 μg/mL to 1.6 μg/mL. In the next step, 50 mg of ninhydrin (Sigma-Aldrich CAS: 485-47-2, Burlington, MA, United States) was dissolved in 10 mL of PBS at pH = 7.4 to give a slightly yellow solution whose concentration was 5 mg/mL. 50 ml of a 5 mg/ml ninhydrin solution was added to 150 ml of gentamicin sulfate solutions to give final concentrations of gentamycin sulfate in the range of 37.50 to 1.20 μg/ml. The prepared samples were vortexed and heated at 95°C for 5 min.

After the elapsed time, the solutions were transferred to an ice bath for 1 min. and immediately examined spectrophotometrically at 418 nm. All measurements were performed in duplicate.

The standard curve for the sulfamethoxazole (S)

11 mg sulfamethoxazole (Sigma-Aldrich, CAS: 723-46-6, Burlington, MA, United States) was accurately reconstituted, which was then dissolved in 10 mL PBS (pH = 7.4) to give a final concentration of 1.1mg/mL. The sulfamethoxazole dilutions in the range of 0.0042-1.1 mg/mL were prepared from the stock solution and analyzed *via* high-performance liquid chromatography using a Waters 2707 instrument (Waters 1525 Binary HPLC Pump) with a vacuum degasser. A detector with a diode array (Waters 2489 UV/Visible Detector) was used to uncover the signal of substance. The reverse-phase column Ascentis® C8 Cat#: 581425-U (25 mm × 4.6 mm, 5 µm) was used for separation. HPLC-grade solvents were employed throughout the analysis: acetonitrile (Merck, UK) with 0.05% trifluoroacetic acid and water with 0.05% trifluoroacetic acid. The sample injection volume was 15 µl, the flow rate was 1 ml/min, and the wavelength for detection was 280 and 254 nm. The retention time for sulfamethoxazole under the described conditions was an average of 13.38 minutes. All measurements were performed in duplicate.

Supplemental figures:

Figure S1

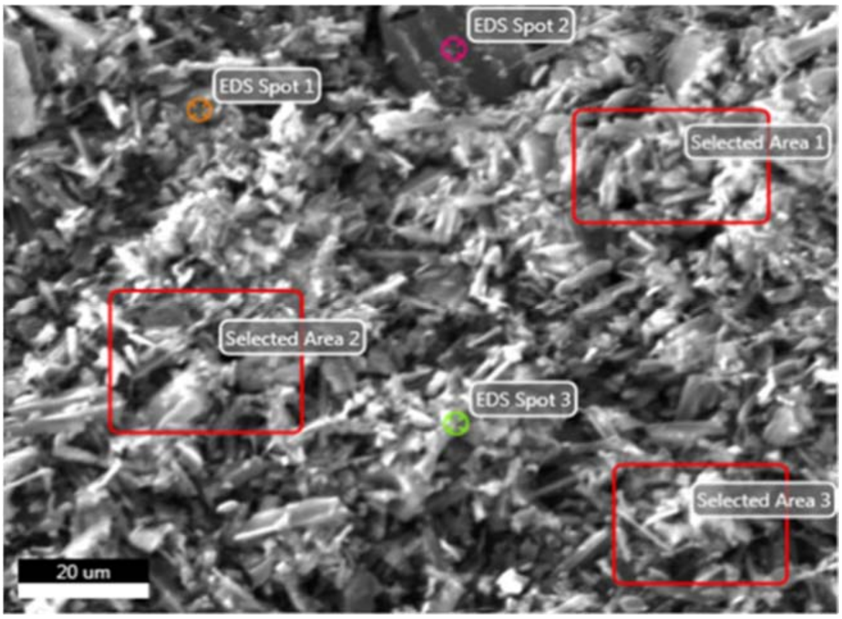

Figure S1. The microscopy picture of the area on PAC surface selected to elemental content analysis. The red windows reveal an area 1-3 on PAC surface. Orange, pink, and green spots show the EDS of 1, 2, and 3, respectively. The areas 1-3 and spots 1-3 of PAC surface were selected to elemental content determination.

Figure S2

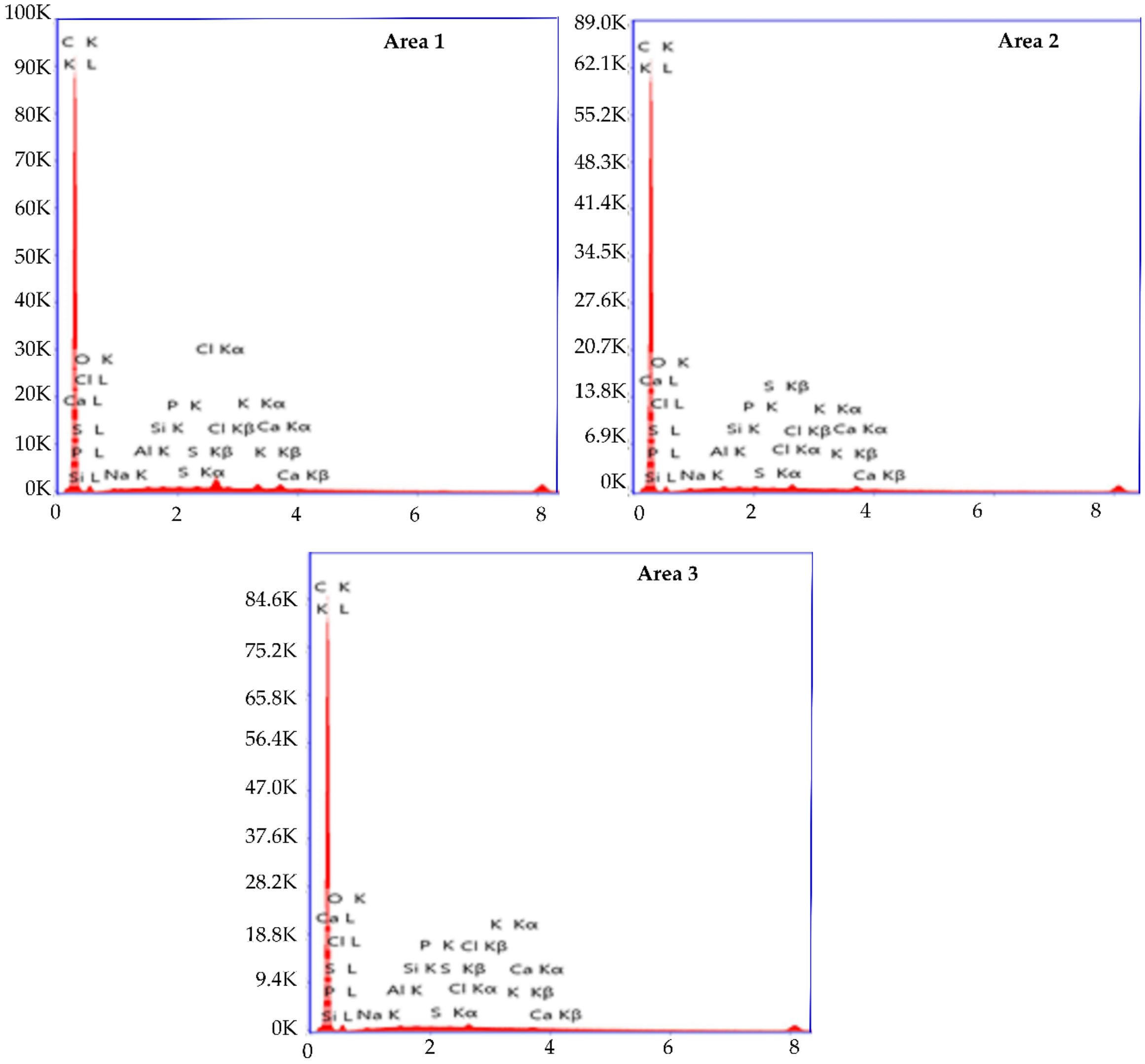
Area 1
100K
90K
80K
70K
60K
50K
40K
30K
20K
10K
0K
0
2
4
6
8
C K
K L
O K
Cl L
Ca L
S L
P L
Si L
Na K
Al K
Si K
P K
S Kα
S Kβ
Cl Kα
Cl Kβ
K Kα
K Kβ
Ca Kα
Ca Kβ
Area 2
89.0K
62.1K
55.2K
48.3K
41.4K
34.5K
27.6K
20.7K
13.8K
6.9K
0K
Area 3
84.6K
75.2K
65.8K
56.4K
47.0K
37.6K
28.2K
18.8K
9.4K
0K

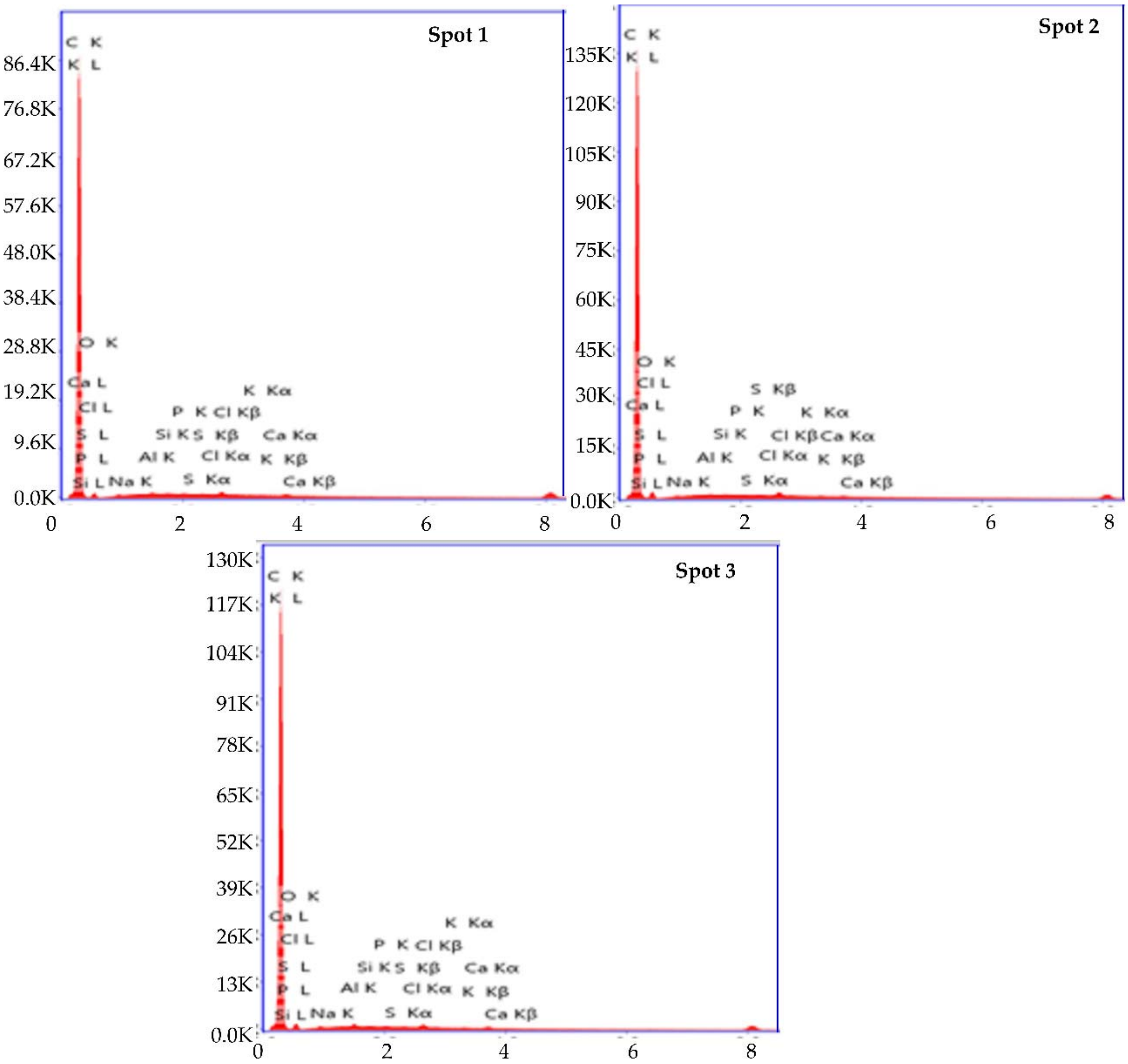

Figure S2. The results of the content of elements for individual selected areas 1-3 and EDS spots 1-3

Figure S3

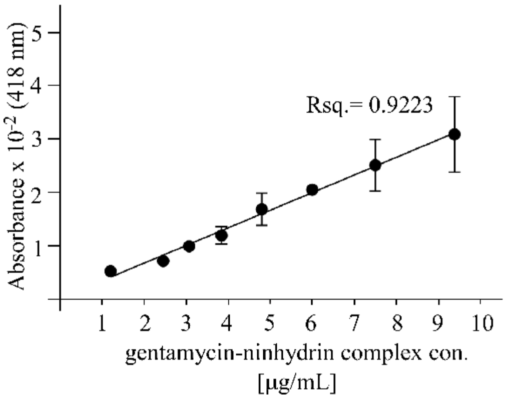

Figure S3. The standard curve for the gentamicin (G)-ninhydrin complex

Figure S4

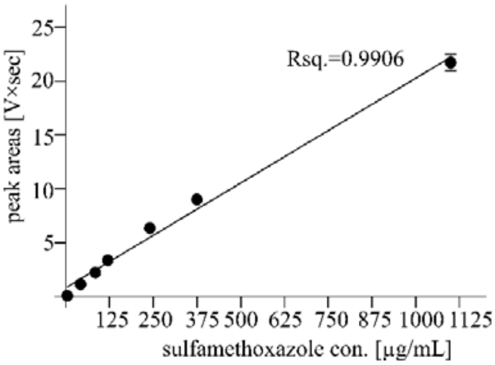

Figure S4 The standard curve for the sulfamethoxazole (S)

Figure S5

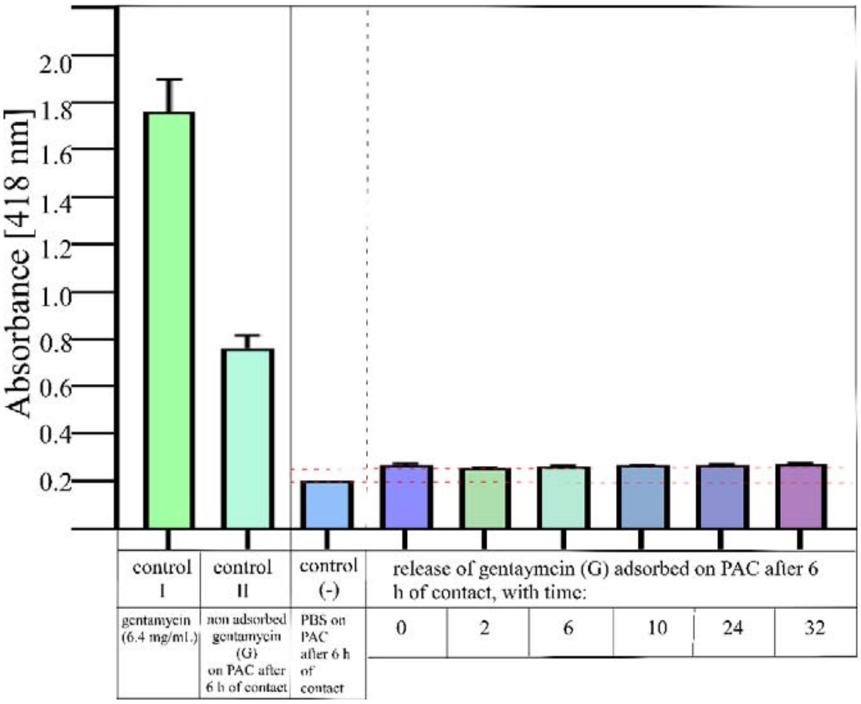

Figures S5 Release of gentamycin sulfate from PAC under aqueous conditions.

Table S1 The results of intelligent eZAF calculations of the content of elements for individually selected areas 1-3 and EDS spots 1-3

	Weight %						Atomic %					
Element	A1	A2	A3	S1	S2	S3	A1	A2	A3	S1	S2	S3
C	92.77	94.04	93.31	94.87	93.2	93.42	95.07	95.84	95.19	96.34	94.99	95.2
O	5.65	4.87	5.84	4.44	6.27	5.9	4.35	3.73	4.47	3.39	4.8	4.52
Na	0.17	0.2	0.13	0.11	0.08	0.21	0.09	0.11	0.07	0.06	0.05	0.11
O	0.05	0.12	0.11	0.09	0.06	0.13	0.02	0.05	0.05	0.04	0.03	0.06
Si	0.07	0.09	0.08	0.06	0.05	0.03	0.03	0.04	0.03	0.03	0.02	0.02

P	0.09	0.12	0.09	0.08	0.05	0.05	0.04	0.05	0.03	0.03	0.02	0.02
S	0.11	0.08	0.09	0.06	0.05	0.03	0.04	0.03	0.03	0.02	0.02	0.01
Cl	0.53	0.21	0.18	0.14	0,16	0.12	0.18	0.07	0.06	0.05	0.05	0.04
K	0.28	0.08	0.08	0.05	0.04	0.03	0.09	0.02	0.02	0.02	0.01	0.01
Ca	0.29	0.2	0.09	0.09	0.05	0.07	0.09	0.06	0.03	0.03	0.01	0.02

A1-3 MEANS AREAS 1-3, S1-3 MEANS SPOTS 1-3

Table S2 Filtration effectiveness of control samples: bacterial cultures *E. coli* and *S. aureus* and activated carbon PAC with LB medium.

CON	A 600 nm	
	Before filtration	After filtration
PAC in LB	0.044	0.045
S. aureus cultured	0.976	0.994
E. coli cultured	0.672	0.616

CON: control samples; A 600 nm: absorbance measured in 600 nm
